# Molecular Characterization of Antibiotic Resistance Determinants in *Klebsiella pneumoniae* Isolates Recovered from Hospital Effluents in the Eastern Cape Province, South Africa

**DOI:** 10.3390/antibiotics12071139

**Published:** 2023-07-01

**Authors:** Joan U. Okafor, Uchechukwu U. Nwodo

**Affiliations:** Patho-Biocatalysis Group (PBG), Department of Biochemistry and Microbiology, University of Fort Hare, Private Bag X1314, Alice 5700, South Africa; okaforjoan14@gmail.com

**Keywords:** hospital, antibiotics resistance, genes, multidrug, *K. pneumoniae*

## Abstract

*Klebsiella pneumoniae* (*K. pneumoniae*) is an opportunistic bacteria responsible for many nosocomial and community-acquired infections. The emergence and spread of antibiotic resistances have resulted in widespread epidemics and endemic dissemination of multidrug-resistant pathogens. A total of 145 *K. pneumoniae* isolates were recovered from hospital wastewater effluents and subjected to antibiogram profiling. Furthermore, the antibiotic resistance determinants were assessed among phenotypic resistant isolates using polymerase chain reaction (PCR). The isolates showed a wide range of antibiotic resistance against 21 selected antibiotics under 11 classes, with the most susceptible shown against imipenem (94.5%) and the most resistant shown against ampicillin (86.2%). The isolates also showed susceptibility to piperacillin/tazobactam (89.0%), ertapenem (87.6%), norfloxacin (86.2%), cefoxitin (86.2%), meropenem (76.6%), doripenem (76.6%), gentamicin (76.6%), chloramphenicol (73.1%), nitrofurantoin (71.7%), ciprofloxacin (79.3%), amikacin (60.7%), and amoxicillin/clavulanic acid (70.4%). Conversely, resistance was also recorded against tetracycline (69%), doxycycline (56.6%), cefuroxime (46.2%), cefotaxime (48.3%), ceftazidime (41.4%). Out of the 32 resistance genes tested, 28 were confirmed, with [*tetA* (58.8%), *tetD* (47.89%), *tetM* (25.2%), *tetB* (5.9%)], [*sul1* (68.4%), *sul1I* (66.6%)], and [*aadA* (62.3%), *strA* (26%), *aac(3)-IIa(aacC2)^a^* (14.4%)] genes having the highest occurrence. Strong significant associations exist among the resistance determinants screened. About 82.7% of the *K. pneumoniae* isolates were multidrug-resistant (MDR) with a multiple antibiotics resistance index (MARI) range of 0.24 to 1.0. A dual presence of the resistant genes among *K. pneumoniae* was also observed to occur more frequently than multiple presences. This study reveals a worrisome presence of multidrug-resistant *K. pneumoniae* isolates and resistance genes in hospital waste effluent, resulting in higher public health risks using untreated surface water for human consumption. As a result, adequate water treatment and monitoring initiatives designed to monitor antimicrobial resistance patterns in the aquatic ecosystem are required.

## 1. Introduction

Hospital wastewaters serve as a channel for disseminating antimicrobial resistance determinants (ARDs) into the environment, such as rivers, streams, and lakes and also serve as a hotspot for their proliferation [1]. Environmental conditions serve as a channel for the spread of antibiotic resistance. The hospital environment is a significant factor in antibiotic resistance distribution [2]. The extensive use of antibiotics in a hospital environment is accountable for disseminating bacteria resistance in such an environment [3]. The likelihood of spreading resistant pathogens from hospitals into the surroundings poses a public health risk if not handled correctly [4]. Human and animal antibiotic misuse promotes the spread of resistant bacteria and environmental resistance genes, endangering public health [5].

There is no sanitation enforcement on wastewater treatment prior to discharge in South Africa. Consequently, this jeopardises the current wastewater system by spreading antibiotic-resistant bacteria and resistance genes into the nearby aquatic environment. Releasing antibiotics-resistant bacteria into the marine ecosystem poses a public health risk. In addition, it has been predicted that between 2015 and 2050, nearly 2.4 million people could die worldwide in low, middle, and high-income countries due to antibiotic-resistant disease-causing microorganisms without persistent attempts to solve the problem [6]. This is particularly alarming in South Africa, where considerations such as insufficient infrastructure, poor sanitation, and a significant percentage of the residents being immune impaired and thus highly vulnerable to infectious illnesses, all lead to an increased risk of antibiotic resistance-related mortality [7].

*Klebsiella pneumoniae* (*K. pneumoniae*) is a multidrug-resistant (MDR) opportunistic pathogen and a substantial cause of hospital-acquired illness common in the South African environment [8]. Multidrug-resistant pathogenic bacteria significantly impact hospital-acquired infection [9]. Modern medicine faces the challenge of increased antibiotic resistance [10]. The therapeutic action of antibiotics against *K. pneumoniae* has been proven abortive due to the development of resistance [11]. *K. pneumoniae* isolated from hospital-acquired infections has high resistance against some commonly used antibiotics such as penicillin, aminoglycosides, tetracyclines, macrolides, lincosamides, folate inhibitors, fluoroquinolones, and phenols. Antibiotic resistance effects, such as infections and death, are thus of great concern.

There are various mechanisms used by *K. pneumoniae* to evade the action of antibiotics. One of the mechanisms of antibiotic resistance is the possession of antibiotic genes called efflux pumps, which modify membrane porosity, the production of inactive enzymes and the alteration of the target location [12]. Bacteria carrying ARDs release their genes into surface waters. Antimicrobial-resistant pathogens and their resistance genes have emerged as a significant water contamination challenge receiving increased global attention. [13]. Therefore, it is pertinent to determine the resistance profile and genes of *K. pneumoniae* in environmental samples such as hospital wastewater to curb the spread of genes into the environment [14]. There is limited information on the antibiogram profile of *K. pneumoniae* isolated from hospital effluents in South Africa. As a result, this study will focus on assessing the resistance patterns and genes of *K. pneumoniae* obtained from hospital wastewater effluents in the Amathole district municipality of the Eastern Cape province of South Africa.

## 2. Results

### 2.1. Confirmation of K. pneumoniae Isolates

Out of 194 presumptive isolates, 168 (86.6%) were confirmed via PCR assay as *Klebsiella* spp. The previously confirmed *Klebsiella* spp. were further delineated into species, and 145/168 (86.3%) were confirmed positive for *K. pneumoniae.* A pictorial representative of *K. pneumoniae* confirmation via PCR amplification is shown in Figures S1 and S2.

### 2.2. Antimicrobial Susceptibility Testing

**Figure 1:**
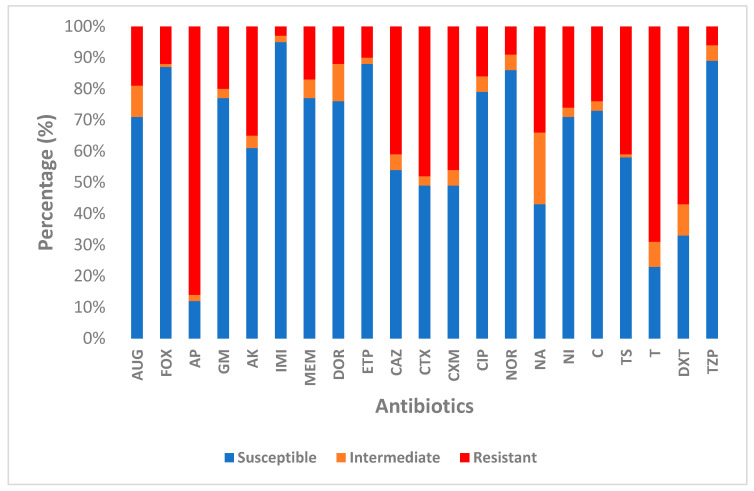
Susceptibility patterns of *K. pneumoniae* isolates.

The antibiotics susceptibility profile of 145 *K. pneumoniae* isolates to 21 antimicrobial agents under 11 different classes was evaluated. Ampicillin had the highest resistance rate at 86%, followed by tetracycline (69%) and doxycycline (57%). Varied resistance to other antibiotics was recorded as follows; cefotaxime (48%), ceftazidime (41%) and trimethoprim-sulfamethoxazole (41%). High sensitivity to imipenem (95%), Piperacillin/tazobactam (89%), Norfloxacin (86%), Ertapenem (88%), Cefoxitin (86%), Ciprofloxacin (79%), Gentamicin (77%), Meropenem (77%), Chloramphenicol (73%), Nitrofurantoin (72%) and Amoxicillin/clavulanic acid (71%) was detected. An isolate (1.45%) was resistant to all antibiotics tested in this study. Figure 1 shows the susceptibility pattern of all *K. pneumoniae* isolates.

### 2.3. Distribution of Antimicrobial Resistance Genes

Simplex, duplex, and multiplex PCR techniques were used to screen 32 genes in *K. pneumoniae* isolates for the presence of tetracycline, beta-lactam, carbapenems, cephems, quinolones, aminoglycosides, phenicol, and sulphonamide resistance. The prevalence and distribution of these genes are shown in Table 1, while a typical gel electrophoresis profile of antimicrobial resistance genes is shown in Figures S3–S5.

Of the 119 resistant tetracycline isolates, 59% possessed the *tetA* gene. Similarly, 48% possessed the *tetD* gene. However, a 25%, 11%, 6% and 3% reduction was observed in *tetM*, *tetK*, *tetB* and *tetC*, respectively. In addition, 17% of the isolates did not harbour any resistance genes. Table 2 shows the dual and multiple resistance genes pattern assessed, with *tetA-tetD* and *tetA-tetM* having the most significant and lowest incidence of 35% and 13%, respectively.

PCR amplification revealed that 17% and 13% of the 142 Beta-lactam resistant isolates harboured *bla_TEM_* and *bla_SHV_* respectively. Table 1 shows the varying prevalence rates of other beta-lactam genes. Among the beta-lactam-resistant isolates, 3% had dual *bla_CTXM-1_-bla_TEM_* and *bla_CTXM-1_-bla_SHV_* resistance genes, while 1% had multiple resistance genes *bla_CTXM-1_–bla_TEM_–bla_OXA-1_* as seen in Table 2. None of the isolates in this study harboured *bla_GES_*, *bla_PER_*, *bla_VEB_*.

Out of the 57 isolates that showed phenotypic resistance to sulphonamides, 68% harboured *sul1*, while 67% had *sul11* genes. A dual resistance pattern (*sul1–sul11*) existed in 60% of the isolates.

The 69 *K. pneumoniae* isolates resistant to aminoglycosides were screened for *strA*, *aadA*, *aac(3)-IIa(aacC2)^a^*, *aph(3)-Ia(aphA1)^a^.* However, *aadA* was the most commonly detected, followed by *strA* and *aac(3)-IIa(aacC2)^a^*, with a prevalence of 26% and 14%, respectively. None of the isolates harboured *aph(3)-Ia(aphA1)^a^*. The most common dual occurrence was *strA-aadA* (15%), followed by *aadA-aac(3)-IIa(aacC2)^a^* (10%). However, multiple resistance pattern *strA-aadA-aac(3)-IIa(aacC2)^a^* was observed to have the most negligible percentage of occurrence (4%).

The most common quinolone resistance gene in the 83 resistant *K. pneumoniae* isolates screened was *qnrA* (16%), followed by *qnrB* (12%). In 41% of the isolates, a dual resistance pattern (*qnrA-qnrB*) was present.

Of the 36 *K. pneumoniae* isolates resistant to phenicols, 31% possessed the *cat11* gene, while 3% possessed the *cat1* gene. All isolates were negative for *cmlA1* resistance genes. Dual and multiple resistance patterns were not observed in any of the isolates.

Five carbapenem resistance genes were screened among the *K. pneumoniae* isolates. The percentage distribution is as follows; *bla_IMP_* (10%), *bla_KPC_* (6%), *bla_NDM-1_* (4%), *bla_VIM_* (2%) and *bla_OXA-48_* (2%). The dual resistance patterns *bla_IMP_–bla_NDM-1_*, *bla_VIM_–bla_KPC_*, and *bla_IMP_–bla_OXA-48_* all had the same occurrence (2%).

### 2.4. MARI Analysis

Multiple antibiotic resistance indexing (MARI) is one of the most straightforward methods for distinguishing antimicrobial resistance trends among environmental isolates and assessing health hazards. It indicates whether the identified isolates are from a high or low-antibiotic-use region. The MARI obtained in this study range between 0.24 and 1.0, as shown in Table 3. A MARI value greater than 0.2 indicates a high risk of source contamination due to antibiotics misuse. Multidrug resistance was observed in 120 (82.7%) *K. pneumoniae* isolates resistant to three or more classes of antibiotics.

### 2.5. Association between the Resistance Genes

The association between resistance genes among *K. pneumoniae* isolates was evaluated and analyzed statistically using the Pearson correlation test (*p* < 0.05), as shown in Table 4. Significant associations existed between some resistance genes harboured in the *K. pneumoniae* isolates.

## 3. Materials and Methods

### 3.1. Study Area Description

**Figure 2:**
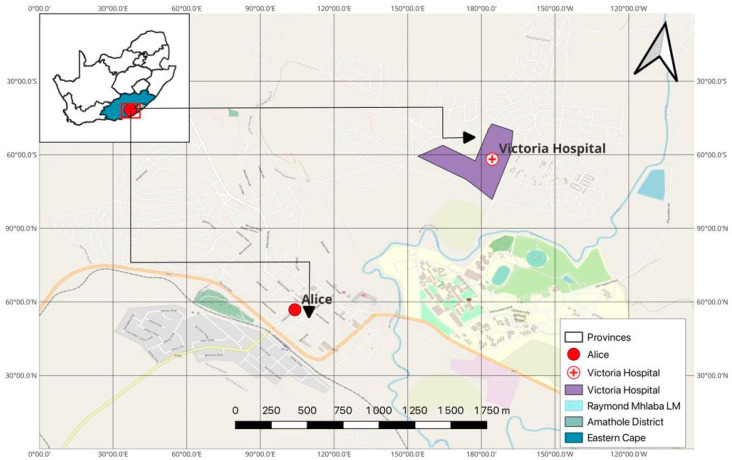
Map of Victoria Hospital sampling point.

The sampling location in this study was Victoria Hospital in the Amathole District Municipality (ADM) in the Eastern Cape Province of South Africa. The residents of these municipalities are mostly employees of community service and agriculture. Most residents visit Victoria Hospital, as it is the largest government hospital in Raymond Mhlaba’s local municipality within ADM. The wastewater effluent from Victoria Hospital is directly discharged into nearby surface water, which residents rely on for agricultural and domestic purposes. A map of the sampling site is shown in Figure 2.

### 3.2. Collection of Samples and Isolation of Presumptive K. pneumoniae

Hospital effluents were aseptically obtained from different sections of Victoria Hospital in triplicates using 1 L sterile glass bottles. The samples were immediately transported to the Applied and Environmental Microbiology Research Group (AEMREG) laboratory at the University of Fort Hare in an ice box for microbiological analyses within 6 h of collection. The wastewater samples were serially diluted 10-fold to 10^−6^, and 100 mL aliquots of each dilution were filtered through a sterile membrane filter of 0.45 μm pore size (Sartorius, Gottingen, Germany). The filtrate was aseptically placed onto MacConkey agar in triplicates. All plates were incubated at 37 °C for 24 h for enumeration as colony-forming per 100 mL (CFU/mL). Afterwards, pink colonies on Mac-Conkey agar show lactose fermentation as presumptive of *Klebsiella* spp. The presumptive isolates were subcultured at 37 °C for 24 h on nutrient agar. The phenotypically pure positive *Klebsiella* spp. colonies were preserved in 20% glycerol stock at −80 °C for further analysis.

### 3.3. Genomic DNA Extraction

Bacterial DNA of the presumptive *Klebsiella* spp. was extracted as previously reported, with slight modifications [15]. Cells from the once-grown isolates on nutrient agar were inoculated into Luria Bertani (LB) broth at 37 °C for 24 h. The cultures were placed into sterile Eppendorf tubes (Biologix, Alondra Camarillo, CA, USA) and centrifuged (LASEC, Hong Kong, China) at 13,5000 rpm for 5 min to wash the cells. The supernatant was decanted, after which 500µL of sterile distilled water was included and vortexed for 2 min using VELP, Scientifica Inc., New York, NY, USA. To lyse the cells, the vortexed cells were placed in a heating block set at 100 °C for 10 min (Techne, London, UK) and subsequently cooled for 5 min on ice. The cell suspension was extracted using a mini-spin micro-centrifuge spun at 13,500 rpm for 5 min. A pipette was used to extract the supernatant, which serves as a template DNA, into sterile Eppendorf tubes for subsequent PCR assays and stored at −20 °C.

### 3.4. Molecular Confirmation of K. pneumoniae

The PCR assay was used to confirm the presumptive isolates using a Mycycler™ Thermal Cycler PCR system (Bio-Rad, T100 thermal cycler, Singapore) under the following conditions: an initial denaturation of 94 °C for 5 min; 35 cycles of 30 s at 94 °C, 45 s annealing at 55 °C, extension at 72 °C for 45 s and a final extension cycle of 72 °C for 10 min. A total reaction mixture of 25 μL consisting of 12.5 μL industrially synthesized master mix (Thermo Scientific, Waltham, MA, USA), 1 μL each of forward and reverse primers (Inqaba Biotech, Mawniya, Nigeria), 5.5 μL PCR grade water,4 μL template DNA. Agarose gel 1.5% and 0.5× TBE buffer, set at 100 V for 45 min, were used to carry out electrophoresis. The gel was stained with 4 μL ethidium bromide. A 100 bp DNA marker (Fermentas, Vilnius, Lithuania) was used to estimate the DNA band, which was then examined with a UV transilluminator (Alliance 4.7, UVITEC, Cambridge, UK). As a positive control, *K. pneumoniae* ATCC 35657 was used. Table 5 lists primers and reaction protocols used to confirm *K. pneumoniae*.

### 3.5. Antibiotics Susceptibility Testing

The antibiotic susceptibility patterns of all *K. pneumonia* isolates were evaluated according to the Kirby–Bauer disk diffusion method using Mueller–Hinton agar medium. The Clinical and Laboratory Standards Institute (CLSI) principles were used for this purpose. The *K. pneumoniae* isolates were subjected to 21 antimicrobial agents under 11 classes commonly used for treating infection caused by *K. pneumoniae*. The antimicrobial agents and their concentration used in this study are Fluoroquinolones: ciprofloxacin (CIP; 5 μg) and norfloxacin (NOR: 30 μg), Aminoglycoside: gentamicin (GM; 10 μg), amikacin (AK; 30 μg), Beta-lactam: amoxicillin/clavulanic acid (AUG; 30 μg), cefoxitin (FOX; 30 μg), ampicillin (AP; 10 μg), Sulfonamides: trimethoprim-sulfamethoxazole (TS; 25 μg); Beta-lactamase inhibitor: piperacillin/tazobactam (TZP), Nitrofurans: nitrofurantoin (NI; 300 μg); Carbapenem: imipenem (IMI; 10 μg), meropenem (MEM; 10 μg), doripenem (DOR; 10 µg), and ertapenem (ETP; 10 µg), Cephalosporins: ceftazidime (CAZ; 30 μg), cefotaxime (CTX; 30 μg) and cefuroxime (CXM; 30 μg); Quinolones: Nalidixic acid (NA; 30 μg), Phenicols: chloramphenicol (C; 30 μg); Tetracyclines: tetracycline (T; 30 μg) and doxycycline (DXT; 30 μg) (MAST, Merseyside, UK). The plates were incubated at 37 °C for 24 h after being inverted for 15 min. The inhibition zones were measured in millimetres and analyzed following the suggested criteria [18]. The isolates were classified as susceptible (S), intermediate (I), and resistant (R). *K. pneumoniae* ATCC 35657 was used as a control strain for the antimicrobial susceptibility tests. Multidrug resistance was evaluated as antimicrobial resistance to at least three or more classes.

### 3.6. Multiple Antibiotics Resistance Index (MARI) Analysis

The MARI was evaluated using the formula: MARI = a/b, where “a” is the number of antibiotics the isolates were resistant to, while “b” is the total number of antibiotics tested [19]. A MARI value greater than 0.2 indicates environmental sources with increased antibiotic misuse [20].

### 3.7. Molecular Detection of Resistance Genes

The presence of resistance genes in phenotypic antibiotics-resistant *K. pneumoniae* was evaluated using the conventional PCR technique with specific synthesized primers (Table S1). The targeted resistance genes include aminoglycosides (*aph(3)-Ia(aphA1)^a^, aac(3)-IIa(aacC2)^a^*, *strA*, *aadA*); quinolones (*qnrA*, *qnrB*, *qnrS*); tetracycline (*tetA*, *tetB*, *tetC*, *tetD*, *tetK*, *tetM*); carbapenems (*bla_VIM_*, *bla_IMP_*, *bla_OXA-48-like_*, *bla_NDM-1_*, *bla_KPC_*); beta-lactams (*bla_GES_*, *bla_VEB_*, *PER*, *bla_SHV_*, *bla_TEM_*, *bla_OXA-1-like_*, *bla_CTX-M-9_*, *bla_CTX-M-2_*, *bla_CTX-M-1_*), sulphonamides (*sul1* and *sul2*), phenicol (*cat1* and *cat11*). Resistance genes were selected according to their prevalence in phenotypically resistant *K. pneumoniae* isolates.

### 3.8. Statistical Analysis

The Statistical Package for Social Sciences (SPSS) IBM version 20 software was used for statistical analysis. Pearson’s correlation test was employed to compare the associations between resistance genes, with *p* < 0.05 as the statistical significance level.

## 4. Discussion

Hospital environments are crucial breeding grounds for antibiotic-resistance microorganisms and resistance genes. Consequently, antimicrobial resistance in hospital effluents is a significant health issue due to the ease of transmission to humans via contaminated water used for many purposes, such as consumption, irrigation, and recreation. Thus, water significantly contributes to the dissemination and consistency of antimicrobial-resistant pathogens and their affiliated potential health threats. Hospital wastewater harbours many bacterial pathogens that are eventually channelled into surface water without proper treatment. The overall prevalence rate of confirmed *K. pneumoniae* isolates obtained in this study was relatively high (86.3%); this could result from antibiotics consumed by hospitalized patients and their subsequent release into the environment. This study’s high detection of *K. pneumoniae* reveals the extent of hospital wastewater effluent contamination by this nosocomial pathogen. Another study in South Africa isolated *K. pneumoniae* from hospital wastewater effluent and recorded a close prevalence rate of 78.8% [21]. Meanwhile, this discovery contradicts other studies with low hospital incidence rates [22,23].

This study observed a high abundance of phenotypic antibiotics-resistant *K. pneumoniae*, which calls for serious concern. The environment’s high level of resistant bacteria represents the uncontrolled and excessive usage of antibiotics [24]. β-lactams are the primary therapeutic option for treating *K. pneumoniae* infections. The high resistance rate of 86.2% against ampicillin, a beta-lactam, is similar to other studies that found increased resistance to ampicillin [25,26,27]. The other beta-lactam antibiotics in this study, amoxicillin/clavulanic acid and cefoxitin, had a relatively low resistance of 19.3% and 12.4%, respectively. Aminoglycosides act by binding to bacterial ribosomes to prevent protein production from commencing. The consumption of aminoglycosides has been curtailed due to long-term use associated with kidney and auditory nerve damage, leading to hearing impairment [28]. In agreement with this finding, *K. pneumoniae* showed a low resistance rate against gentamicin and amikacin compared to previous studies [29,30]. This study’s low resistance to aminoglycosides could be due to its reduced usage. Similar to this study, a resistance rate of 38.6% against amikacin was reported among carbapenem-resistant *Klebsiella pneumoniae* isolates in China [31].

Chloramphenicol inhibits protein synthesis by binding to the ribosomes. Moreover, chloramphenicol is associated with developing aplastic anaemia in humans, hence the ban on its usage [32]. This study’s resistance rate to chloramphenicol (23.5%), similar to another study’s reported rate in Bangladesh [33], could likewise be due to its prohibition. In this study, *K. pneumoniae* isolates showed a reduced resistance rate to carbapenems. However, an average percentage of isolates were reported to be resistant to ertapenem (47.6%), imipenem (42.9%), and meropenem (42.9%) in another study [34]. Similar to a study in Saudi Arabia, resistance to imipenem (3.4%) was the weakest [26]. Meanwhile, cephalosporin resistance was discovered in close to half of the isolates in this study, including ceftazidime (41.4%), cefotaxime (48.3%), and cefuroxime (46.2%). This is higher than the findings from another study that isolated *K. pneumoniae* from cow milk [30]. Rabbani et al. [33] reported a moderate resistance rate against cefotaxime (45%) and ceftazidime (38%), similar to this study. Moreover, Manikandan and Amsath observed a similar resistance rate to ceftazidime (45.8%) [35]. Moderate resistance to tetracycline (69.0%), doxycycline (56.6%), trimethoprim-sulfamethoxazole (40.7%), nalidixic acid (33.8%) were observed in this study. The increased resistance rate of these antibiotics classes in this study could be attributed to common drug abuse in health care.

Carbapenems have the highest susceptibility rate in this study, with varied susceptibility rates against imipenem (94.5%), ertapenem (87.6%), doripenem (76.6%) and meropenem (76.6%). Other studies have also recorded high susceptibility rates of 85.64% against meropenem and 63.61% for imipenem [25,36]. In a similar report, *K. pneumoniae* isolates showed high susceptibility to imipenem [21,33]. Low resistance rates of 35% and 34% to ertapenem and meropenem, which contradicts our findings, have been reported elsewhere in South Africa [37]. The susceptibility rate against fluoroquinolones (ciprofloxacin 79.3% and norfloxacin 86.2%) and Nitrofurans (Nitrofurantoin 71.7%) is similar to another report [10]. In this study, aminoglycosides (amikacin and gentamicin) exhibited a high susceptibility rate, in line with an earlier study [38].

The widespread usage of antimicrobial agents has resulted in a high incidence of MDR *K. pneumoniae* strains [39]. The present research reveals multidrug resistance to various antimicrobials ranging between three and ten antibiotics classes in 82.7% of *K. pneumoniae* isolates with a MARI range of 0.14–1.0. The MARI range indicates a significant environmental source of contamination. The high prevalence of MDR *K. pneumoniae* in this study also demonstrates the potential dangers of *K. pneumoniae* spreading from hospital sources. A similar occurrence of MDR was reported in other studies [10,20,26,40,41].

The current study used a PCR-based technique to characterize antimicrobial resistance genes in *K. pneumoniae* isolates from hospital effluents. Many studies have revealed that antibiotics resistance genes (ARGs) are spread due to polluted environmental sources [42,43]. Some essential resistance genes in clinical and environmental settings have been initially documented in *K. pneumoniae* [44,45]. Carbapenem was the drug of last resort for treating MDR infections until recently, when its efficacy was diminished due to its widespread misuse. One of the essential antibiotic resistance mechanisms is carbapenemase production. However, our study revealed a low percentage of *K. pneumoniae* isolates harbouring carbapenem genes, with varied rates as follows; *bla_IMP_* (9.8%), *bla_KPC_* (5.8%), *bla_NDM-1_* (3.9%), *bla_VIM_* (1.9%), *bla_OXA-48_* (1.9%). This study’s low occurrence of carbapenem genes could be due to the earlier report on the decrease in resistance level to carbapenem antibiotics, as shown in Figure 1. Our findings are consistent with other studies that show a low overall prevalence of carbapenem genes [37,46]. However, this study’s observable low incidence of carbapenem genes contradicts a report in another study where *bla_OXA_* was abundant in hospital wastewater [47]. Meanwhile, in another study, the rates for *bla_NDM-1_* and *bla_OXA-48_* were 33.3% and 66.7%, respectively [34]. The presence of *bla_IMP_* in this study is comparable to research that examined carbapenem genes in clinical samples [48]. Findings on carbapenem genes in Gram-negative strains have been compiled across the globe, such as in Kenya [49], Uganda [50], Egypt [51], South Africa [52], Iraq [53], and the Philippines [54] with varying prevalence.

Sulphonamides inhibit dihydropteroate synthetase, an enzyme that uses para-aminobenzoic acid (PABA) as a substrate to produce folic acid. Sulphonamide genes (*sul1* and *sul11*) were detected, with *sulI* (68.4%) and *sulI1* (66.6%) common in our isolates. In 60% of the isolates, both genes were found, which indicates a possible abuse of the sulphonamides class of antibiotics. This study’s high abundance of *sul11* is similar to another study in South Africa [55]. The most commonly used antibiotics are beta-lactams due to their low toxicity and use in treating many infections. Nonetheless, the gravity of their resistance cannot be overemphasized. They have minimal side effects and are utilized to treat various infectious diseases. *Bla_TEM_* (16.90%) is the most common beta-lactamase gene in this study, while *bla_SHV_* (12.67%) is more common than *bla_CTXM-1_* (9.15%). The least prevalent beta-lactam gene is *bla_CTXM-2_*_,_ with an occurrence of 0.7%. Folake et al. reported *bla_TEM_* as the most abundant beta-lactam gene [55]. Other studies have noted several beta-lactamase genes and reported them in bacteria isolates [55,56,57].

With the advent of tetracycline, tetracycline-resistant bacteria became widespread in the environment. Out of six tetracycline genes screened, the *tetA* (58.82%) gene had the highest occurrence, followed by *tetD* (47.89%) and *tetM* (25.21%). Other varied resistance rates are as follows; *tetK* (10.92%), *tetB* (5.88% and *tetC* (3.36%). The abundance of these genes in the aquatic ecosystem may be due to effluent influx and uncontrolled sewage discharge. Other studies have reported tetracycline genes in marine ecosystems [55,56,58].

Among the aminoylsocide-resistant *K. pneumoniae* isolates, the most prevalent resistant gene is *aadA* (62.3%), while *strA* and *aac(3)-IIa(aacC2)^a^* has resistance rate of 26% and 14.4%, respectively. A higher prevalence rate of 43.1% was reported for *aac(3)-IIa(aacC2)^a^* in another study in Spain. The combination of *strA-aadA* was observed in 15% of isolates, while *aadA–aac(3)-11a(aac(2)^a^* was found in 10% of isolates. However, *strA–aadA–aac(3)-11a(aac(2)^a^* had the least rate of occurrence at 4%. In this study, *qnrA* was the most dominant quinolone gene, found in 15.6% of *K. pneumoniae* isolates with phenotypic resistance to quinolones. In comparison, *qnrB* was found in 12% of the isolates. No isolate in this study harboured *qnrS*. Notwithstanding this, in another study, *qnrS* was the most frequently observed quinolone gene [34].

Numerous phenicol genes are abundant in the environment. In this study, *K. pneumoniae* isolates resistant to chloramphenicol were screened for *cat1*, *cat11 and cmlA*. A similarly low rate of *cat11* and *cat1* was reported in another study, which could have resulted from the restrictions on the abuse of chloramphenicol [56]. None of the isolated strains harboured the *cmla* gene, and dual chloramphenicol resistance genes were not recorded in this study. There were strong statistical associations between *K. pneumoniae* resistance genes, as shown in Table 4. Most of the resistance determinants of *K. pneumoniae* isolates showed a strong and significant association.

In conclusion, there is an alarming presence of MDR *K. pneumoniae* isolates in hospital wastewater effluent in the study area. However, our findings indicated a possible link between multidrug resistance development in hospital sewage and the disposal of untreated hospital waste. Nonetheless, an attempt must be made to avert the unhygienic disposal of waste harbouring antibiotics into the environment via hospital drainage channels. This necessitates immediate intervention through a proper surveillance program as well as adequate treatment of wastewater before discharge to surface water and subsequent human consumption. *K. pneumoniae* isolates showed high sensitivity to carbapenems and harboured few carbapenem resistance genes; thus, carbapenem could be used for treating *K. pneumoniae* infections. This study revealed a noticeable collaboration of phenotypic resistance with the genotypic expression of resistance genes. Single and multiple resistance genes were also reported among the phenotypic-resistant *K. pneumoniae* isolates. Future studies should focus on genotypic resistance gene evaluation. We anticipate that this study’s findings will help establish a foundation for future scientific research in South Africa regarding the burden of MDR *K. pneumoniae* in the environment and its public health consequences.

## Figures and Tables

**Table 1:** Distribution of antimicrobial resistance genes among *K. pneumoniae* isolates.

Antibiotics Resistance Genes	Total Positive (%)
Beta-Lactam (*n* = 142 isolates screened)	
*bla_TEM_*	24 (16.90)
*bla_SHV_*	18 (12.67)
*bla_OXA-1-like_*	2 (1.40)
*bla_CTX-M-1_*	13 (9.15)
*bla_CTX-M-2_*	1 (0.70)
*bla_CTX-M-9_*	4 (2.8)
Tetracycline (*n* = 119 isolates screened)	
*tetA*	70 (58.82)
*tetB*	7 (5.88)
*tetC*	4 (3.36)
*tetD*	57 (47.89)
*tetK*	13 (10.92)
*tetM*	30 (25.21)
Carbapenem (*n* = 51 tested)	
*IMP*	5 (9.8)
*VIM*	1 (1.9)
*KPC*	3 (5.8)
*NDM-1*	2 (3.9)
*OXA-48*	1 (1.9)
Aminoglycoside (*n* = 69 tested)	
*strA*	18 (26.0)
*aadA*	43 (62.3)
*aac(3)-IIa(aacC2)^a^*	10 (14.4)
Sulphonamides (*n* = 57 tested)	
*sul1*	39 (68.4)
*sul2*	38 (66.6)
Quinolones (*n* = 83 tested)	
*qnrA*	13 (15.6)
*qnrB*	10 (12.0)
Phenicol (*n* = 36 tested)	
*cat1*	2 (5.6)
*cat11*	11 (30.5)

**Table 2:** Patterns of dual and multiple resistance determinants tested in the antimicrobial-resistant *K. pneumoniae* isolates.

Antimicrobial Class	Antimicrobial Resistance Determinants Pattern	Total (%)
Beta-lactam	*bla_CTXM-1_–bla_TEM_*	4 (3)
	*bla_CTXM-1_–bla_TEM_–bla_OXA-1_*	2 (1)
	*bla_CTXM-1_–bla_SHV_*	4 (3)
Carbapenem	*bla_IMP_–bla_NDM-1_*	1 (2)
	*bla_VIM_–bla_KPC_*	1 (2)
	*bla_IMP_–bla_OXA-48_*	1 (2)
Aminoglycosides	*strA-aadA*	10 (15)
	*strA–aadA–aac(3)-11a(aac(2)^a^*	3 (4)
	*aadA–aac(3)-11a(aac(2)^a^*	7 (10)
Sulfonamides	*sul1-sul11*	34 (60)
Quinolones	*qnrA-qnrB*	34 (41)
Tetracycline	*tetA-tetC*	4 (3)
	*tetA-tetB*	4 (3)
	*tetA-tetD*	41 (35)
	*tetA-tetK*	12 (10)
	*tetA-tetM*	16 (13)
	*tetD-tetM*	7 (6)
	*tetB-tetD*	1 (1)
	*tetA-tetB-tetK*	1 (1)
	*tetA-tetD-tetM*	9 (8)
	*tetA-tetD-tetK*	9 (8)
	*tetA-tetB-tetD*	1 (1)
	*tetD-tetK-tetM*	1 (1)
	*tetA-tetC-tetM*	2 (2)
	*tetA-tetC-tetD*	1 (1)
	*tetA-tetD-tetK-tetM*	4 (3)

**Table 3:** MARI analysis.

Number of Antibiotics	Frequency (%)	MARI
1	3 (2)	0.05
2	4 (2.8)	0.09
3	18 (12.6)	0.14
4	14 (9.8)	0.19
5	12 (8.5)	0.24
6	18 (12.6)	0.29
7	13 (9.2)	0.33
8	8 (5.6)	0.38
9	15 (10.6)	0.43
10	8 (5.6)	0.47
11	9 (6.3)	0.52
12	9 (6.3)	0.57
13	2 (1.4)	0.62
14	3 (2.1)	0.67
15	3 (2.1)	0.71
17	1 (0.7)	0.80
19	1 (0.7)	0.90
20	1 (0.7)	0.95
21	1 (0.7)	1.0

**Table 4:** Antimicrobial resistance genes association.

Genes	*SHV*	*OXA-1*	*CTXM-1*	*CTXM-2*	*CTXM-9*	*tetA*	*tetB*	*tetC*	*tetD*	*tetK*	*tetM*	*IMP*	*VIM*	*KPC*	*NDM*	*OXA-48*	*strA*	*aadA*	*aaC2*	*sul1*	*sul11*	*qnrA*	*qnrB*	*cat1*	*cat11*
*TEM*	−	+++	−	+++	+++	+++	++	+++	+++	−	−	+++	+++	+++	+++	+++	−	+	+	−	−	−	+	+++	+
*SHV*		+++	−	+++	++	+++	+	+	+++	−	−	+	+++	++	+++	+++	−	++	−	++	++	−	−	+++	−
*OXA-1*			++	−	−	+++	−	−	+++	++	+++	−	−	−	−	−	+++	+++	+	+++	+++	++	+	−	+
*CTXM-1*				++	+	+++	−	+	+++	−	+	−	++	+	++	++	−	+++	−	+++	++	−	−	++	−
*CTXM-2*					−	+++	−	−	+++	++	+++	−	−	−	−	−	+++	+++	+	+++	+++	++	+	−	++
*CTXM-9*						+++	−	−	+++	+	+++	−	−	−	−	−	++	+++	−	+++	+++	+	−	−	−
*tetA*							+++	+++	−	+++	+++	+++	+++	+++	+++	+++	+++	+	+++	++	++	+++	+++	+++	+++
*tetB*								−	+++	−	+++	−	−	−	−	−	+	+++	−	+++	+++	−	−	−	−
*tetC*									−	+	+++	−	−	−	−	−	++	+++	−	+++	+++	+	−	−	−
*tetD*										+++	++	+++	+++	+++	+++	+++	+++	−	+++	−	−	+++	+++	+++	+++
*tetK*											+	−	++	+	++	++	−	+++	−	+++	+++	−	−	++	−
*tetM*												+++	+++	+++	+++	+++	−	−	++	−	−	+	++	+++	++
*IMP*													−	−	−	−	+	+++	_	+++	+++	−	−	−	−
*VIM*														−	−	−	+++	+++	+	+++	+++	++	+	−	++
*KPC*															−	−	+++	+++	−	+++	+++	+	−	−	−
*NDM*																−	+++	+++	+	+++	+++	++	+	−	+
*OXA-48*																	+++	+++	+++	+++	++	++	+	−	++
*strA*																		++	−	++	++		−	++	−
*aadA*																			+++	−	−	+++	+++	+++	+++
*aaC2*																				+++	+++	−	−	+	−
*sul1*																					−	+++	+++	+++	+++
*sul11*																						++	+++	+++	+++
*qnrA*																							−	++	−
*qnrB*																								+	−
*cat1*																									++

The associations of antimicrobial resistance genes at the *p* < 0.05 level are shown. The levels of significance of the association were as follows: −, *p* > 0.05; +, 0.05 ≥ *p* ≥ 0.01; ++, 0.01 ≥ *p* ≥ 0.001; and +++, 0.001 ≥ *p*.

**Table 5:** Primer sequences and PCR cycling conditions.

Target Strain	Target Gene	Primer Sequence (5^1^–3^1^)	PCR Cycling Condition	Product Size (bp)	Reference
*Klebsiella* genus	*gyrA*	F:CGCGTACTATACGCCATGAACGTA R:ACCGTTGATCACTTCGGTCAGG	‘‘94 °C (5 min), 94 °C (30 s), 55 °C (45 s), 72 °C (45 s), 72 °C (10 min) × 35 cycles”	441	[16]
*K. pneumoniae*	*magA*	F:ATTTGAAGAGGTTGCAAACGAT R: TTCACTCTGAAGTTTTCTTGTGTTC	‘‘94 °C (5 min), 94 °C (30 s), 55 °C (30 s), 72 °C (45 s), 72 °C (10 min) × 30 cycles”	130	[17]

## Data Availability

Further information will be provided on request.

## Supplementary Materials

The following supporting information can be downloaded at: https://www.mdpi.com/article/10.3390/antibiotics12071139/s1, Table S1: Primer sequences of targeted resistance genes with their respective amplicon sizes and PCR cycling conditions. References [59,60,61,62,63,64,65,66] are cited in the supplementary materials; Figure S1: Agarose gel electrophoresis showing confirmed Klebsiella spp. (*gyrA*). Lane M: 100 bp DNA ladder, lane 1: Positive control (*K. pneumonie* ATCC 35657), lane 2: Negative control lane 3–10 positive *Klebsiella* spp. Isolates; Figure S2: Agarose gel electrophoresis showing confirmed *Klebsiella pneumonie* (*magA*). Lane M: 100 bp DNA ladder, lane 1: Positive control (*K. pneumonie* ATCC 35657), lane 2: Negative control, lane 3–11 positive *K. pneumonie* isolates; Figure S3: A representative gel electrophoresis profile of some antimicrobial resistance genes of *K. pneumonie* isolates. Lanes M and 12: molecular weight marker (Thermo Scientific 100 bp DNA ladder), lane 1: negative control, lane 2: *tetA* (201bp), lane 3: *tetB*, (659bp), lane 4: *tetC* (418 bp), lane 5: *tetD* (787 bp), lane 6: *tetK* (750 bp), lane 7: *sul1* (722 bp), lane 8: *sul11* (256 bp), lane 9: *aac(3)-IIa(aacC2)^a^* (428 bp), lane 10: *strA* (348 bp), and lane 11: *aadA* (318 bp); Figure S4: A representative gel electrophoresis profile of some antimicrobial resistance genes of *K. pneumonie* isolates. Lanes M: molecular weight marker (Thermo Scientific 100 bp DNA ladder), lane 1: negative control, lane 2: *bla_SHV_* (713bp), lane 3: *bla_oxa-1-like_* (564bp), lane 4: *bla_IMP_* (139 bp), lane 5: *bla_KPC_* (301 bp), lane 6: *bla_NDM-1_* (251 bp), lane 7: *bla_OXA-48_* (281 bp), lane 8: *bla_TEM_* (800 bp), lane 9: *bla_CTXM-9_* (561 bp); Figure S5: A representative gel electrophoresis profile of some antimicrobial resistance genes of *K. pneumonie* isolates. Lane M: molecular weight marker (Thermo Scientific 100 bp DNA ladder), lanes 1 and 2: *bla_CTXM-1_* (688bp), lanes 3 and 4: *qnrB* (469bp), lane 5: *cat11* (543bp), lane 6: *bla_VIM_* (390 bp), lane 7: *cat1* (320 bp), lane 8: negative control.
