# Supplementary material for: Molecular Characterization of Antibiotic Resistance Determinants in Klebsiella pneumoniae Isolates Recovered from Hospital Effluents in the Eastern Cape Province, South Africa
Source: Antibiotics (Basel). 2023 Jul 1;12(7):1139. doi: 10.3390/antibiotics12071139 (PMC10376002; doi:10.3390/antibiotics12071139)
Supplement: Supplementary file 1 [file antibiotics-12-01139-s001.zip › antibiotics-2451359-supplementary.pdf]

Table S1: Primer sequences of targeted resistance genes with their respective amplicon sizes and PCR cycling conditions.

Gene classification	Target gene	Primer Sequence (5^1-3^1)	PCR cycling condition	Product size (bp)	Reference
Beta-lactam			''94 °C (10mins), 94 °C (40sec), 60 °C (40sec), 72 °C (1min), 72 °C (7mins) x 30 cycles"		[59]
bla$_{CTX\text{-}M\ 1}$	*MultiCTXMGp1_for*<br>*MultiCTXMGp1-2_rev*	TTAGGAARTGTGCCGCTGYA[b]<br>CGATATCGTTGGTGGTRCCAT[b]		688	
bla$_{CTX\text{-}M\text{-}2}$	*MultiCTXMGp2_for*<br>*MultiCTXMGp1-2_rev*	CGTTAACGGCACGATGAC<br>CGATATCGTTGGTGGTRCCAT[b]	''94 °C (10mins), 94 °C (40sec), 60 °C (40sec), 72 °C (1min), 72 °C (7mins) x 30 cycles"	404	[59]
bla$_{CTX\text{-}M\text{-}9}$	*MultiCTXMGp9_for*<br>*MultiCTXMGp9_rev*	TCAAGCCTGCCGATCTGGT<br>TGATTCTCGCCGCTGAAG	''94 °C (10mins), 94 °C (40sec), 60 °C (40sec), 72 °C (1min), 72 °C (7mins) x 30 cycles"	561	[59]
bla$_{OXA\text{-}1\text{-}like}$	*MultiTSO-O_for*<br>*MultiTSO-O_rev*	GGCACCAGATTCAACTTTCAAG<br>GACCCCAAGTTTCCTGTAAGTG	''94 °C (10mins), 94 °C (40sec), 60 °C (40sec), 72 °C (1min), 72 °C (7mins) x 30 cycles"	564	[59]
bla$_{SHV}$	*MultiTSO-S_for*<br>*MultiTSO-S_rev*	AGCCGCTTGAGCAAATTAAAC<br>ATCCCGCAGATAAATCACCAC	''94 °C (10mins), 94 °C (40sec), 60 °C (40sec), 72 °C (1min), 72 °C (7mins) x 30 cycles"	713	[59]
bla$_{TEM}$	*MultiTSO-T_for*<br>*MultiTSO-T_rev*	CATTTCCGTGTCGCCCTTATTC<br>CGTTCATCCATAGTTGCCTGAC	''94 °C (10mins), 94 °C (40sec), 60 °C	800	[59]

			(40sec), 72 °C (1min), 72 °C (7mins) x 30 cycles”		
bla$_{VEB}$	*MultiVEB_for*<br>*MultiVEB_rev*	CATTTCCCGATGCAAAGCGT<br>CGAAGTTTCTTTGGACTCTG	‘’94 °C (10mins), 94 °C (40sec), 60 °C (40sec), 72 °C (1min), 72 °C (7mins) x 30 cycles”	648	[59]
bla$_{PER}$	*MultiPER_for*<br>*MultiPER_rev*	GCTCCGATAATGAAAGCGT<br>TTCGGCTTGACTCGGCTGA	‘’94 °C (10mins), 94 °C (40sec), 60 °C (40sec), 72 °C (1min), 72 °C (7mins) x 30 cycles”	520	[59]
bla$_{GES}$	*MultiGES_for*<br>*MultiGES_rev*	AGTCGGCTAGACCGGAAAG<br>TTTGTCCGTGCTCAGGAT	‘’94 °C (10mins), 94 °C (40sec), 57 °C (40sec), 72 °C (1min), 72 °C (7mins) x 30 cycles”	399	[59]
**Carbapenem**<br>*bla*$_{VIM}$	*MultiVIM_for*[c]<br>*MultiVIM_rev*[c]	GATGGTGTTTGGTCGCATA<br>CGAATGCGCAGCACCAG	‘’94 °C (10mins), 94 °C (40sec), 55 °C (40sec), 72 °C (1min), 72 °C (7mins) x 30 cycles”	390	[59]
bla$_{IMP}$	*MultiIMP_for*<br>*MultiIMP_rev*	TTGACACTCCATTTACDG[b]<br>GATYGAGAATTAAGCCACYCT[b]	‘’94 °C (10mins), 94 °C (40sec), 55 °C (40sec), 72 °C (1min),	139	[59]

			72 °C (7mins) x 30 cycles”		
bla$_{OXA\text{-}48\text{-}like}$	*bla*$_{OXA\text{-}48\text{-}like\text{-}F}$<br>*bla*$_{OXA\text{-}48\text{-}like\text{-}R}$	TTGGTGGCATCGATTATCGG<br>GAGC ACTT CTTT TGTG ATGG C	‘’94 °C (10mins), 94 °C (40sec), 57 °C (40sec), 72 °C (1min), 72 °C (7mins) x 30 cycles”	744	[59]
bla$_{NDM\text{-}1}$	*bla*$_{NDM\text{-}1\text{-}F}$<br>*bla*$_{NDM\text{-}1\text{-}R}$	AAAACGGCAAGAAAAAGCAG<br>AAAACGGCAAGAAAAAGCAG	‘’94 °C (10mins), 94 °C (40sec), 57 °C (40sec), 72 °C (1min), 72 °C (7mins) x 30 cycles”	251	[59]
bla$_{KPC}$	*bla*$_{KPC\text{-}F}$<br>*bla*$_{KPC\text{-}R}$	AAAACGGCAAGAAAAAGCAG<br>AAAACGGCAAGAAAAAGCAG	‘’94 °C (10mins), 94 °C (40sec), 55 °C (40sec), 72 °C (1min), 72 °C (7mins) x 30 cycles”	301	[59]
**Quinolones**	*qnrA*	ATTTCTCACGCCAGGATTTG<br>GATCGGCAAAGGTTAGGTCA	‘’94 °C (10mins), 94 °C (45sec), 53 °C (45sec), 72 °C (1min), 72 °C (7mins) x 32 cycles”	516	[60]
	qnrB	GATCGTGAAAGCCAGAAAGG<br>ACGATGCCTGGTAGTTGTCC	‘’94 °C (10mins), 94 °C (45sec), 53 °C (45sec), 72 °C (1min), 72 °C (7mins) x 32 cycles”	469	[60]
	qnrS	ACGACATTCGTCAACTGCAA<br>TAAATTGGCACCCTGTAGGC	‘’94 °C (10mins), 94 °C (45sec), 53 °C (45sec), 72 °C (1min),	417	[60]

			72 °C (7mins) x 32 cycles”		
**Aminoglycosides**	*aac(3)-IIa(aacC2)*[a]	F:CGGAAGGCAATAACGGAG<br>R:TCGAACAGGTAGCACTGAG	’’94 °C (5mins), 94 °C (30sec), 50 °C (30sec), 72 °C (1.30mins), 72 °C (5mins) x 30 cycles”	428	[61]
	aadA	F:GTGGATGGCGGCCTGAAGCC<br>R:AATGCCCAGTCGGCAGCG	’’94 °C (4mins), 94 °C (45sec), 50 °C (45sec), 72 °C (45secs), 72 °C (7mins) x 30 cycles”	318	[62]
	strA	F:CTTGGTGATAACGGCAATTC<br>R:CCAATCGCAGATAGAAGGC	’’94 °C (4mins), 94 °C (45sec), 50 °C (45sec), 72 °C (45secs), 72 °C (7mins) x 30 cycles”	348	[62]
	aph(3)-Ia(aphA1)[a]	F:ATGGGCTCGCGATAATGTC<br>R:CTCACCGAGGCAGTTCCAT	’’94 °C (5mins), 94 °C (30sec), 50 °C (30sec), 72 °C (1.30mins), 72 °C (5mins) x 30 cycles”	600	[61]
**Sulphonamides**	*sul1*	F:TTCGGCATTCTGAATCTCAC<br>R:ATGATCTAACCCTCGGTCTC	’’94 °C (5mins), 94 °C (1min), 55 °C (1min), 72 °C (5mins), 72 °C (5mins) x 35 cycles”	722	[61]
	sul11	F:CGGCATCGTCAACATAACC<br>R:GTGTGCGGATGAAGTCAG	’’94 °C (5mins), 94 °C (30sec), 50 °C (30sec), 72 °C (1.5mins), 72 °C (5mins) x 30 cycles”	256	[63]

**Phenicol**	*cat1*	F:AGTTGCTCAATGTACCTATAACC<br>R:TTGTAATTCATTAAGCATTCTGCC	''94 °C (5mins), 94 °C (30sec), 50 °C (30sec), 72 °C (1.5mins), 72 °C (5mins) x 30 cycles"	320	[61]
	cat11	F:ACACTTTGCCCTTTATCGTC<br>R:TGAAAGCCATCACATACTGC	''94 °C (5mins), 94 °C (30sec), 50 °C (30sec), 72 °C (1.5mins), 72 °C (5mins) x 30 cycles"	543	[61]
	cmlA1	F: CACCAATCATGACCAAG<br>R: GGCATCACTCGGCATGGACATG	''94 °C (5mins), 94 °C (30sec), 50 °C (30sec), 72°C (1.5mins), 72 °C (5mins) x 30 cycles"	115	[64]
**Tetracycline**	*tetA*	F: GCTACATCCTGCTTGCCTTC<br>R:CATAGATCGCCGTGAAGAGG	''94 °C (5mins), 94 °C (1min), 55 °C (1min), 72 °C (1.5mins), 72 °C (5mins) x 35 cycles"	201	[65]
	tetB	F: TTGGTTAGGGGCAAGTTTTG<br>R:GTAATGGGCCAATAACACCG	''94 °C (5mins), 94 °C (1min), 55 °C (1min), 72 °C (1.5mins), 72 °C (5mins) x 35 cycles"	659	[65]
	tetC	F: CTTGAGAGCCTTCAACCCAG<br>R: ATGGTCGTCATCTACCTGCC	''94 °C (5mins), 94 °C (1min), 55 °C (1min), 72 °C (1.5mins), 72 °C (5mins) x 35 cycles"	418	[65]
	tetD	F:AAACCATTACGGCATTCTGC<br>R:GACCGGATACACCATCCATC	''94 °C (5mins), 94 °C (1min), 55 °C (1min), 72 °C (1.5mins), 72 °C (5mins) x 35 cycles"	787	[65]

	tetK	F:GTAGCGACAATAGGTAATAGT R:GTAGTGACAATAAACCTCCTA	‘’94 °C (5mins), 94 °C (1min), 55 °C (1min), 72 °C (1.5mins), 72 °C (5mins) x 35 cycles”	760	[66]
	tetM	F: AGTGGAGCGATTACAGAA R:CATATGTCCTGGCGTGTCTA	‘’94 °C (5mins), 94 °C (1min), 55 °C (1min), 72 °C (1.5mins), 72 °C (5mins) x 35 cycles”	158	[66]

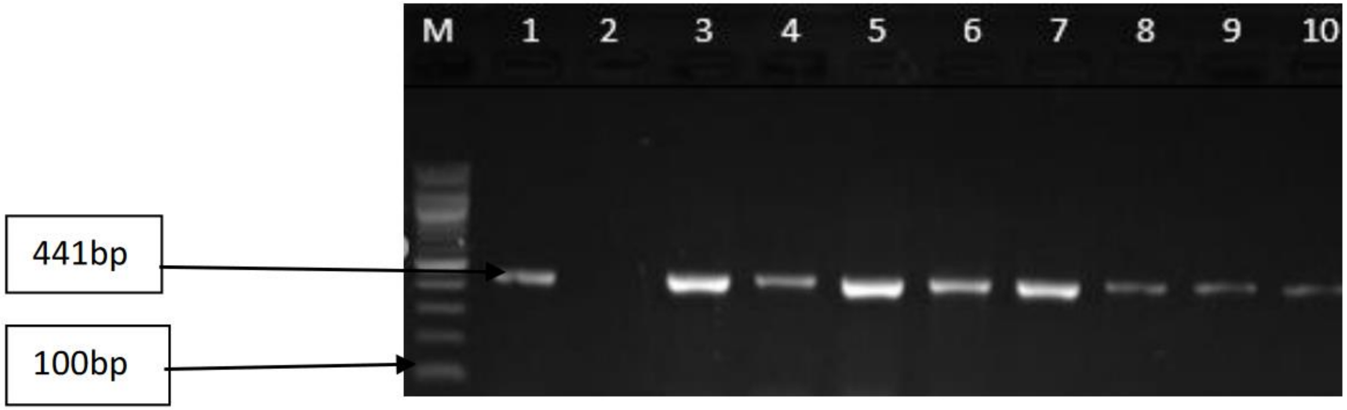

Figure S1. Agarose gel electrophoresis showing confirmed Klebsiella spp (*gyrA*). Lane M:100 bp DNA ladder, lane 1: Positive control (*K. pneumonie* ATCC 35657), lane 2: Negative control lane 3-10 positive *Klebsiella* spp. isolates.

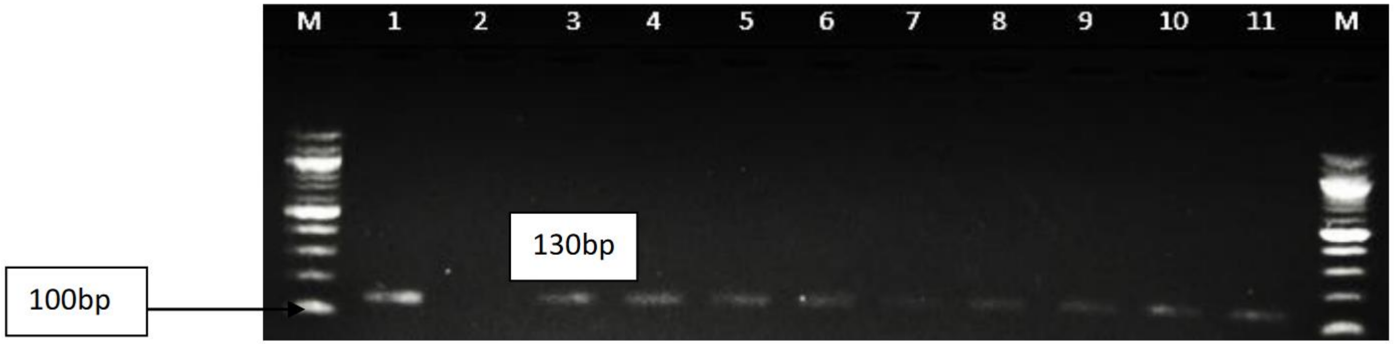

Figure S2. Agarose gel electrophoresis showing confirmed *Klebsiella pneumonie* (*magA*). Lane M:100 bp DNA ladder, lane 1: Positive control (*K. pneumonie* ATCC 35657), lane 2: Negative control, lane 3-11 positive *K. pneumonie* isolates.

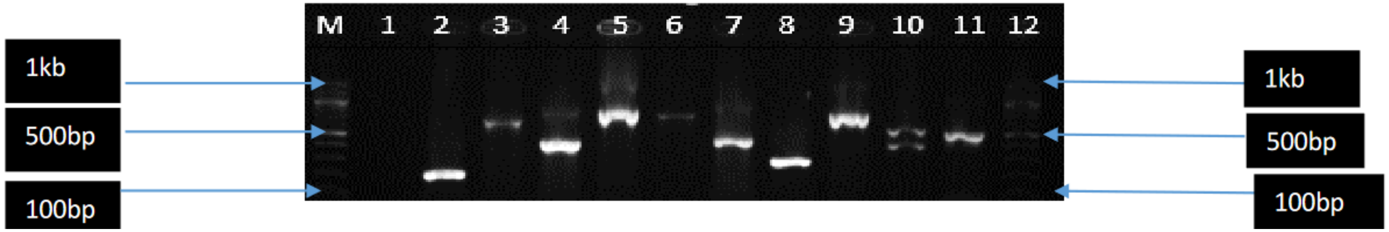

Figure S3. A representative gel electrophoresis profile of some antimicrobial resistance genes of *K. pneumonie* isolates. Lanes M and 12: molecular weight marker (Thermo Scientific 100 bp DNA ladder), lane 1: negative control, lane 2: *tetA* (201bp), lane 3: *tetB*, (659bp), lane 4: *tetC* (418 bp), lane 5: *tetD* (787 bp), lane 6: *tetK* (750 bp), lane 7: sul1 (722 bp), lane 8: sul11 (256 bp), lane 9: *aac(3)-IIa(aacC2)*[a] (428 bp), lane 10: strA(348 bp), and lane 11: aadA (318 bp).

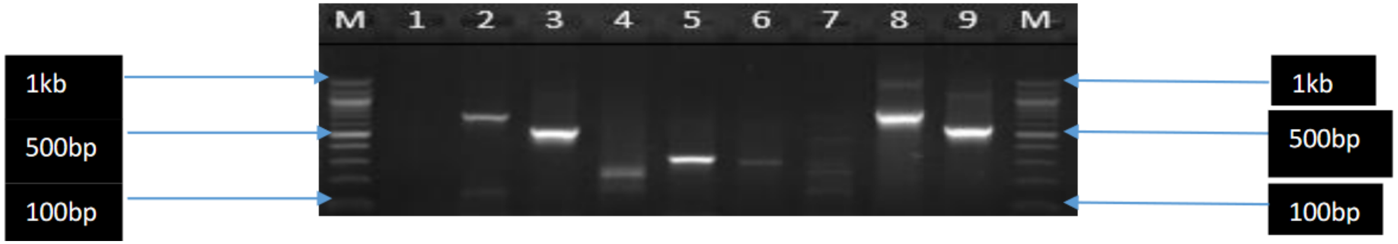

Figure S4. A representative gel electrophoresis profile of some antimicrobial resistance genes of *K. pneumonie* isolates. Lanes M: molecular weight marker (Thermo Scientific 100 bp DNA ladder), lane 1: negative control, lane 2: bla$_{SHV}$ (713bp), lane 3: bla$_{oxa\text{-}1\text{-}like}$ (564bp), lane 4: bla$_{IMP}$ (139 bp), lane 5: bla$_{KPC}$ (301 bp), lane 6: bla$_{NDM\text{-}1}$(251 bp*)*, lane 7: bla$_{OXA\text{-}48}$ (281 bp), lane 8: bla$_{TEM}$(800 bp), lane 9: bla$_{CTXM\text{-}9}$ (561 bp).

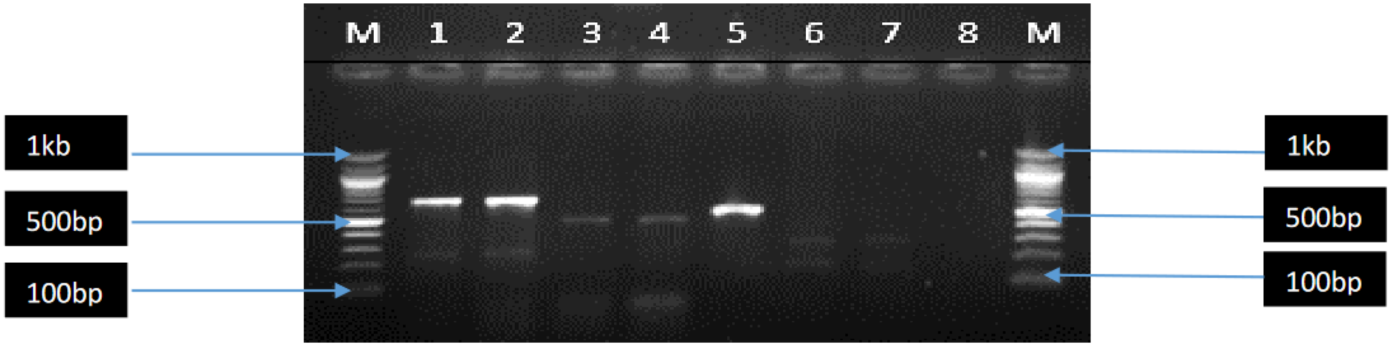

Figure S5. A representative gel electrophoresis profile of some antimicrobial resistance genes of *K. pneumonie* isolates. Lane M: molecular weight marker (Thermo Scientific 100 bp DNA ladder), lanes 1 and 2: bla_{CTXM-1} (688bp), lanes 3 and 4: QnrB (469bp), lane 5: Cat11 (543bp), lane 6: bla_{VIM} (390 bp), lane 7: Cat1 (320 bp), lane 8:negative control.
